# Supplementary material for: Relevant Serum Endoplasmic Reticulum Stress Biomarkers in Type 2 Diabetes and Its Complications: A Systematic Review and Meta-Analysis
Source: Antioxidants (Basel). 2024 Dec 19;13(12):1564. doi: 10.3390/antiox13121564 (PMC11673038; doi:10.3390/antiox13121564)
Supplement: Supplementary file 1 [file antioxidants-13-01564-s001.zip › antioxidants-3310996-supplementary.pdf]

SUPPLEMENTARY DATA

Supplementary file 1. Query for search

PubMed Query: 845 results

(ER stress OR endoplasmic reticulum stress OR CHOP OR PERK OR XBP1 OR Glucose-regulated protein OR GRP OR Grp78 OR Grp94 OR Grp170 OR heat shock protein OR HSP OR HSP12A OR HSP70 OR HSP72 OR HSP25 OR HSP27 OR HSP60 OR HSP60 OR HSP90 OR HSP47 OR HSPA8 OR HSP12A OR HSP65 OR chaperon OR chaperones OR Peroxiredoxin OR Peroxiredoxin OR PRX1 OR PRX2 OR PRX4 OR PRX6 OR secretagogin) AND (diabetes OR glycemia OR insulin resistance) AND (biomarkers OR markers) AND (serum OR plasma OR blood)

Scopus Query: 106 results

(er AND stress OR endoplasmic AND reticulum AND stress OR chop OR perk OR xbp1 OR glucose-regulated AND protein OR grp OR grp78 OR grp94 OR grp170 OR heat AND shock AND protein OR hsp OR hsp12a OR hsp70 OR hsp72 OR hsp25 OR hsp27 OR hsp60 OR hsp60 OR hsp90 OR hsp47 OR hspa8 OR hsp12a OR hsp65 OR chaperon OR chaperones OR peroxiredoxin OR peroxiredoxin OR prx1 OR prx2 OR prx4 OR prx6 OR secretagogin) AND (diabetes OR glycemia OR insulin AND resistance)

Web of Science Query: **248 results**

(ER stress OR endoplasmic AND reticulum AND stress OR chop OR perk OR xbp1 OR glucose-regulated AND protein OR grp OR grp78 OR grp94 OR grp170 OR heat AND shock AND protein OR hsp OR hsp12a OR hsp70 OR hsp72 OR hsp25 OR hsp27 OR hsp60 OR hsp60 OR hsp90 OR hsp47 OR hspa8 OR hsp12a OR hsp65 OR chaperon OR chaperones OR peroxiredoxin OR peroxiredoxin OR prx1 OR prx2 OR prx4 OR prx6 OR secretagogin) AND (diabetes OR glycemia OR insulin AND resistance)

TOTAL n=1199

Supplementary file 2. Reasons for the exclusion of studies that were not eligible.

Author, year	Reason of exclusion
Calabrese, 2007	This study measured Hsp60 and Hsp70 in circulating lymphocytes
Calabrese, 2012	This study measured Hsp70 in circulating lymphocytes
Ding, 2022	This study does not include healthy subjects
Anklam, 2021	This study focused on the relationship of eHSP72 with menopause
Garamvölgyi, 2015	This study measure HSPA1A in gestational diabetes
Gruden, 2018	This study was performed in subjects with T1D
Imatoh, 2009	The serum HSP60 levels were not detectable
Islam, 2014	This study does not include diabetic subjects
Kapalla, 2005	This study measured Hsp90 in circulating leucocytes
Morteza, 2013	This study does not include healthy subjects
Nargesi, 2015	This study does not include healthy subjects
Pengiram, 2009	This study does not include diabetic subjects
El-Horany, 2017	This study measure HSP72 in urine

-No ERS markers quantification in serum/plasma n=4

-No inclusion of controls or diabetic subjects n=5

-Study subjects with different type of diabetes or condition n=3

-Indetectable levels of ERS markers in serum/plasma n=1
